# Characterization of Grape Pomace Extract Microcapsules: The Influence of Carbohydrate Co-Coating on the Stabilization of Goat Whey Protein as a Primary Coating

**DOI:** 10.3390/foods13091346

**Published:** 2024-04-27

**Authors:** Gabriela Perković, Josipa Martinović, Gordana Šelo, Ana Bucić-Kojić, Mirela Planinić, Rita Ambrus

**Affiliations:** 1 Faculty of Food Technology Osijek, Josip Juraj Strossmayer University of Osijek, F. Kuhača 18, HR-31 000 Osijek, Croatia; gperkovic@ptfos.hr (G.P.); gselo@ptfos.hr (G.Š.); abucic@ptfos.hr (A.B.-K.); 2 Faculty of Pharmacy, Institute of Pharmaceutical Technology and Regulatory Affairs, University of Szeged, H-6720 Szeged, Hungary

**Keywords:** grape pomace, encapsulation, spray drying, goat whey protein, trehalose, sucrose, xylose, maltodextrin, microcapsule characterization, phenolic compounds

## Abstract

Both grape pomace and whey are waste products from the food industry that are rich in valuable ingredients. The utilization of these two by-products is becoming increasingly possible as consumer awareness of upcycling increases. The biological activities of grape pomace extract (GPE) are diverse and depend on its bioavailability, which is influenced by processes in the digestive system. In this work, goat whey protein (GW) was used as the primary coating to protect the phenolic compounds of GPE during the spray drying process. In addition, trehalose (T), sucrose (S), xylose (X), and maltodextrin (MD) were added to the goat whey proteins as co-coatings and protein stabilizers. All spray drying experiments resulted in microcapsules (MC) with a high encapsulation efficiency (77.6–95.5%) and yield (91.5–99.0%) and almost 100% recovery of phenolic compounds during the release test. For *o*-coumaric acid, the GW-coated microcapsules (MC) showed a bioavailability index of up to 731.23%. A semi-crystalline structure and hydrophilicity were characteristics of the MC coated with 10% T, S, X, or 5% MD. GW alone or in combination with T, S, MD, or X proved to be a promising carrier for polyphenols from grape pomace extract and ensured good bioavailability of these natural antioxidants.

## 1. Introduction

Grapes are one of the most cultivated crops worldwide [1,2,3]. The majority of grapes planted are meant to be used for wine production [4]. Twenty to thirty percent of the grapes used to produce wine are classified as grape pomace, which includes leftover pulp, seeds, stems, and grape skins [2]. Owing to its high content of phenolic compounds, lipids, proteins, dietary fibers, and a variety of biological activities such as antioxidant [5], antiproliferative [6], anti-inflammatory [7], cardioprotective [8,9], and antimicrobial activity [10], grape pomace has been the subject of numerous studies. The great interest in researching the beneficial effects of phenolic compounds derived from grapes, i.e., those contained in wine, was triggered by the discovery of the French paradox thirty years ago [11]. On the other hand, the realization that only about 30% of these phenols from grapes pass into the wine, while the rest of the phenolic substances remain in the waste, has also brought grape pomace into the focus of scientific research. The growing awareness of the importance of upcycling and the great potential of utilizing by-products that are rich in phenolic compounds for the production of fortified products is a major contributing factor. By isolating the phenolic substances from the original source, they are additionally exposed to the negative effects of oxygen, heat, and light, which can compromise the positive effects on human health that can be achieved through their consumption. Therefore, the importance of encapsulation is also recognized as a technique that can be used to improve the shelf life and stability of various extracts that are rich in phenolic substances obtained from different natural sources. One of the most popular methods for encapsulating phenolic compounds is spray drying, which uses various coating materials to convert a liquid phenolic extract directly into a product in the form of microcapsules (MCs) [12]. The main advantages of the spray drying process are its easy scalability, flexibility, low processing costs, and the low water content in the product, which ensures the controlled release of the entrapped bioactive substances [13]. As proven in numerous studies, the characteristics of MCs are influenced by the spray drying conditions, such as inlet air temperature, the feed flow, the air flow, the configuration of the drying chamber and the cyclone itself, the diameter of the nozzle, and the composition of the feed, more precisely the liquid feed concentration. The use of a carrier material enables better flowability, compressibility, and compatibility of the MC [14]. The most widely utilized coatings, either separately or in combination, for protecting phenolic compounds are gum arabic [15,16,17], maltodextrin [12,16,18,19,20] and whey proteins [20,21]. 

Whey is the yellow–green liquid fraction that remains after cheese and casein production. It was considered useless, disposed of in nature without prior treatment, and ignored for many years. Studies a few years ago unearthed the wealth concealed by this dairy industry by-product. Actually, the composition of whey includes a wide variety of proteins, the presence of which has been linked to a wide range of biological activities and, consequently, a wide range of beneficial effects on human health [22]. Whey protein can be used as a texture modifier, thickener, carrier/vehicle, gelling agent, surfactant, foaming agent, and water binder, as well as an effective encapsulation system for active food and drug components, improving their solubility, transport, dispersibility, and bioaccessibility [23]. Whey can therefore be used for the development of new products such as edible films, hydrogels, various coatings, and micro- and nanoparticles. 

What makes goat’s milk preferable to cow’s milk is its better digestibility, alkalinity, buffering capacity [24], its better anti-inflammatory effects on the intestines [25], and its lower allergenic potential [26]. Goat’s milk and whey have already proven to be successful as coating materials in the spray drying process for the protection of various bacteria [27,28]. So far, it has been found that the use of this coating produces smaller microparticles with a pronounced ability to retain the active component and that no agglomeration and clumping occur due to the absence of agglutinin in the resulting MC [28].

Disaccharide sugars are often used as protein stabilizers during the spray drying process of whey or whey protein, where their presence can prevent denaturation of the protein and preserve the original structure and functionality of the protein as much as possible. When sugar is added as a protective component, its presence causes spray-dried MC to become amorphous and spherical with a wrinkled or folded surface [29]. According to reports by Cui et al. [30], trehalose and sucrose can use their hydrogen bonding connections to shield protein molecules from dehydration during the spray drying process. A similar protective effect against protein dehydration stress was also observed during freeze drying. When a protein solution is freeze-dried in the presence of sugar, amorphous sugar matrices are formed in which protein molecules can be embedded. It has been proposed that the interactions between protein and sugar molecules during dehydration and storage are prevented by the embedding protein molecules in the amorphous sugar matrix [31]. The potential functional and health benefits of the interactions between whey protein and polysaccharides, particularly the formation of soluble food-grade complexes, are captivating. Some of the potential applications of protein–polysaccharide complexes include fat mimesis, encapsulation, protection, and delivery of bioactive compounds during digestion and modification of colloidal structures in foods [32].

To our knowledge, this is the first paper that investigates the properties of spray-dried grape pomace extract with the addition of goat whey protein (GW) as coating material and the addition of protein stabilizers, i.e., trehalose (T), sucrose (S), xylose (X), and maltodextrin (MD) as co-coating materials. The aim of this study was to encapsulate Cabernet Sauvignon grape pomace extract (GPE) using the spray drying technique and the mentioned coating materials. The additional objective was to investigate the influence of the coating materials on the structure and properties of the MCs as well as the release and bioaccessibility index (BI) of phenolic compounds from the MCs of grape pomace extract.

## 2. Materials and Methods

### 2.1. Chemicals and Reagents

The coatings used were 78.6% goat whey protein (GW) from Carrington Farms, (Closter, NJ, USA), D(+)-Sucrose 99+% (S) from Acros Organics, D(+)-Xylose (X) from AppliChem GmbH (Darmstadt, Germany), trehalose (T) from Hayashibara doo (Nagase group, Tokyo, Japan), and maltodextrin (dextrose equivalent 4–7) (MD) from Sigma Aldrich (Saint Louis, MO, USA). Folin–Ciocalteu’s phenol reagent was purchased from CPAchem (Bogomilovo, Bulgaria), 96% ethanol (p.a.) from Lab Expert (Shenzhen, Guangdong, China). Glacial acetic acid (99.5%) and methanol HPLC grade were purchased from Macron Fine Chemicals (Gliwice, Poland). Sodium carbonate (anhydrous, p.a.) was purchased from T.T.T. (Sveta Nedjelja, Croatia). The standards for the UHPLC analysis of phenolic compounds were obtained from Sigma Aldrich (Saint Louis, MO, USA), Extrasynthese (Genay, France), Acros Organics (Geel, Belgium), and Applihem (Darmstadt, Germany). The chemicals used for simulated digestion (enzymes, bile extract) were obtained from Sigma Aldrich (Saint Louis, MO, USA). The salts used for the preparation of solutions and buffers were obtained from Acros Organics (Geel, Belgium), Gram Mol (Zagreb, Croatia), and Kemika (Zagreb, Croatia).

### 2.2. Grape Pomace

The winery Erdut (Erdut, Croatia, 2017 harvest) provided the grape pomace of the Cabernet Sauvignon (*Vitis vinifera* L.) variety grape (GP) that was left over after vinification. The grape pomace was air-dried to a dry matter content greater than 90% and ground to a particle size of ≤1 mm using an ultracentrifugal mill (Retsch ZM 200, Haan, Germany) immediately before extraction.

### 2.3. Grape Pomace Extract Preparation

Phenolic compound extraction from the GP was performed in a shaking water bath (Julabo SW-23, Seelbach, Germany) at 80 °C and 200 rpm for 2 h. The ratio of the prepared grape pomace sample and 50% aqueous ethanol solution was 1 g:40 mL. After extraction, the samples were centrifuged at 11,000 rcf for 10 min (Z 326 K, Hermle Labortechnik GmbH, Wehingen, Germany). The resulting liquid phenol-rich extract was evaporated in a rotary evaporator (Büchi, R-210, Flawil, Germany) at 48 mbar and 50 °C to half of the initial volume in order to reduce the ethanol content in the extract due to the limitations of the spray-drying device. The volume of ethanol removed was replaced by an equal volume of redistilled water, and this extract (GPE) was used to prepare the encapsulation solution (spray drying feed).

### 2.4. Encapsulation via Spray Drying

After the preparation of GPE, a feed mixture for encapsulation was prepared by mixing GPE and GW in a ratio of (mass of dry matter of the extract):(mass of dry matter of the coating) = 1:2.5 (*w*/*w*). When a carbohydrate co-coating (protein stabilizer) was added, part of the GW was replaced by the corresponding mass of the co-coating, which was between 2.5% and 30% depending on the stabilizer used (Table 1). Feed homogenization was carried out using a magnetic stirrer (SMHS-6, Witeg, Germany) at 50 °C and 600 rpm for 10 min. Spray drying was performed using a Büchi B-290 mini spray dryer (Flawil, Switzerland). The feed was spray-dried under the following conditions: inlet temperature 173 °C, feed flow 7 mL/min, nozzle diameter 15 µm.

### 2.5. Total Phenolic Content Determination 

First, the sample was prepared according to the instructions given by Tolun et al. [33]. In brief, 3 mL of a solution of ethanol/glacial acetic acid/water (50:8:42, *v*/*v*/*v*) was added to 15 mg of the MC, mixed using a vortex mixer, and then filtered through a 0.45 μm PTFE filter. The total phenolic content (TPC) was determined using the Folin–Ciocalteu method described by Waterhouse, with modifications. Briefly, 3160 µL of distilled water was mixed with 40 µL of prepared sample and 200 µL of Folin–Ciocalteu reagent. After an 8 min incubation period, 600 µL of 20% (*w*/*v*) sodium carbonate was added, and the mixture was further incubated at 40 °C. After 30 min, the absorbance was measured at 765 nm against a blank containing distilled water instead of the sample. The results were expressed as mass of gallic acid equivalents per mass of MC dry matter (mg_GAE_/g_db_).

### 2.6. Surface Phenolic Content Determination 

For the determination of surface phenolic compounds (SPC), the sample was prepared according to Tolun et al. [33] as follows: 3 mL of ethanol/methanol (1:1, *v*/*v*) solution was added to 24 mg of MC, and after 5 min, the sample was filtered through a 0.45 μm PTFE filter. This was followed by the determination of the content of phenolic compounds according to the Folin–Ciocalteu method described in Section 2.5. The results were expressed as mass of gallic acid equivalents per mass of MC dry matter (mgGAE/gdb).

### 2.7. Encapsulation Efficiency

The encapsulation efficiency (EE) was calculated according to Vu et al. [18] using Equation (1):

(1) $$EE\left(\%\right)=\frac{C_{TPC}-C_{SPC}}{C_{TPC}}\times 100$$

where *C*_TPC_ is the mass fraction of the total phenolic content in the MCs (mg_GAE_/g_db_) and *C*_SPC_ is the mass fraction of the surface phenolic content in the MCs (mg_GAE_/g_db_).

### 2.8. Moisture and Dry Matter Content

As per Kelly et al. [34], the moisture and dry matter content of the MC and coating materials were ascertained by employing the thermogravimetric method using a halogen moisture analyzer (Mettler Toledo HR73, Columbus, OH, USA). A sample of 0.15 g was weighed on an aluminum plate. The standard method of drying at 105 °C with switch-off criteria 5 (that is, until the mass loss was less than 1 mg for 140 s) was then applied. The moisture and dry matter values of the MC were calculated according to Equations (2) and (3):

(2) $$Moisture\left(\%\right)=\frac{m_{B}-m_{A}}{m_{B}}\times 100$$

(3) $$Dry\;matter\;(\%)=\frac{m_{A}}{m_{B}}\times 100$$

where *m*_A_ is mass of the sample after drying and *m*_B_ is mass of the sample before drying.

### 2.9. Encapsulation Yield

The encapsulation yield (Y) was determined using Equation (4) in accordance with Vu et al. [18].

(4) $$Y(\%)=\frac{total\;mass\;of\;dry\;matter\;of\;the\;powder\left(g\right)}{total\;mass\;of\;dry\;matter\;in\;the\;feed\left(CSE+coating\right)\left(g\right)}\times 100$$

### 2.10. Bulk Density and Tapped Density 

With some modifications, the bulk density (BD) for the microcapsules and coating materials was calculated using the method described by Boyano-Orozco et al. [35]. A total of 1 g of the sample was put into a 25 mL beaker, and using the Equation (5), the bulk density was determined as the mass of the MC divided by the volume of the MC read on the beaker.

(5) $$Bulk\;density\left(g/{cm}^{3}\right)=\frac{sample\;mass}{sample\;volume}$$

The tapped density (TD) was determined using an AutoTap device (Anton Paar, Graz, Austria), according to Boyano-Orozco et al. [35] with modifications. Using Equation (6), the value for the tapped density was determined as the ratio of MC mass to MC volume following 1250 taps of the MC beaker.

(6) $$Tapped\;density\;\left(g/{cm}^{3}\right)=\frac{sample\;mass}{sample\;volume\;after\;1250\;taps}$$

### 2.11. Hausner Ratio and Carr Index

The compressibility properties of the powder were expressed using the Hausner ratio (HR) and the Carr index (CI), which were calculated according to Kalušević et al. [16] using Equations (7) and (8).

(7) $$Hausner\;ratio\;\left(-\right)=\frac{bulked\;density}{tapped\;density}$$

(8) $$Carr\;index\left(\%\right)=\frac{bulked\;density-tapped\;density}{bulked\;density}\times 100$$

### 2.12. Particle Size Distribution

The particle size of the produced MCs was measured via dynamic light scattering using a Malvern Mastersizer 2000 (Malvern Instrument, Malvern, UK) with water used as the dispersing agent and the refractive index set to 1.62.

### 2.13. Determination of the Solubility of the Microcapsules

The MC solubility was determined according to Lee et al. [36] with minor modifications. Petri dishes were washed, then dried in a dryer (Memmert UFE 500, Schwabachu, Germany) at 105 °C for 1 h, and after cooling, they were weighed together with the lid. Then, 0.1 g of MCs was weighed into a previously weighed 50 mL Falcon test tube and, 10 mL of redistilled water was added. The contents of the test tube were mixed using a vortex mixer (DLAB SCIENTIFIC MX-S, Beijing, China), and the test tube was placed in a water bath (Witeg WSB-30, Wertheim am Main, Germany) at 60 °C for 30 min. At the end of the incubation, the Falcon test tubes with the samples were cooled in cold water and then centrifuged (Hermle Z 326 K, Gosheim, Germany) for 10 min at 11,000 rcf. The supernatant was decanted into a Petri dish, where it was dried for 3 h at 105 °C in an electric dryer (Memmert UFE 500, Schwabachu, Germany). After drying, the Petri dishes with the samples were placed in a desiccator to cool for 1 h and then weighed to determine the mass of the dissolved MC. In addition, the Falcon test tube with sediment was weighed to determine the mass of the remaining (swollen) sediment. Based on the obtained results, the values of the water solubility index (WSI), water adsorption index (WAI), and swelling capacity (SP) were calculated according to Equations (9)–(11).

(9) $$WSI\left(\%\right)=\frac{m_{supernatant\;after\;drying}\left(g\right)}{m_{dry\;basis\;powder}\left(g\right)}\times 100$$

(10) $$WAI\;\left(-\right)=\frac{m_{residue\;left\;after\;centrifugation}\left(g\right)}{m_{dry\;basis\;powder}\left(g\right)}$$

(11) $$SP\;\left(-\right)=\frac{m_{residue\;left\;after\;centrifugation}\left(g\right)}{m_{dry\;basis\;powder}\left(g\right)\times \left(1-\frac{WSI\;\left(\%\right)}{100}\right)}$$

### 2.14. Contact Angle and Polarity 

Wu’s harmonic mean equation (2007) was utilized to calculate the contact angle (θ) of the MC pressed into a disk using an OCA 20 Optical Contact Angle Measuring System (Dataphysics, Filderstadt, Germany). A Specac hydraulic press (Specac Inc., Orpington, UK) was used for the pressing, and it had a pressing diameter of 13 mm and a pressing force of 1 t for 60 s. Using the sessile drop method, the contact angle between water and diiodomethane was measured in the system. This allowed for the determination of the interfacial tension between the polar (*γ*^P^_s_) and dispersive (*γ*^d^_s_) components of the polymers. According to Equation (12), the MC surface free energy (γ*) was calculated as the sum of these quantities.

(12) $$\gamma^{*}=\gamma_{s}^{P}+\gamma_{s}^{d}$$

In accordance with the ratio of the polar surface energy to the MC surface free energy, the polarity of the MCs was determined according to Equation (13).

(13) $$Polarity\left(\%\right)=\frac{\gamma_{s}^{P}}{\gamma^{*}}\times 100$$

### 2.15. X-ray Powder Diffraction

An X-ray powder diffraction system (XPRD) (BRUKER D8 Advance diffractometer, Karlsruhe, Germany) was used to examine the crystalline structure of the coatings and MCs. The samples were exposed to Cu Kα radiation (λ = 1.5406 Å) and were scanned using a VÅNTEC-1 detector at 40 kV and 40 mA at intervals of 3–40 2θ. DIFFRAC plus EVA software Version 13.0.0.1 (Karlsruhe, Germany) was used for smoothing, Kα2-stripping, and background removal as part of the results evaluation process.

### 2.16. Differential Scanning Calorimetry

Using differential scanning calorimetry (DSC) (Mettler Toledo 821e DSC; Mettler Inc., Schwerzenbach, Switzerland), the thermal behavior of the MCs and coating material samples was examined. About 3–5 mg of MC was accurately weighed into DSC sample pans, which were hermetically sealed and lid pierced. An empty pan was used as a reference in an inert atmosphere under a constant argon purge of 150 mL/min. The samples were examined in the temperature range of 25–300 °C at a heating rate of 10 °C/min.

### 2.17. Scanning Electron Microscopy

Scanning electron microscopy (SEM) (Hitachi S4700, Hitachi Scientific Ltd., Tokyo, Japan) was used to examine the MC morphology. Using a sputter coater (Bio-Rad SC 502, VG Microtech, Uckfield, UK), MCs were coated with a thin layer of gold–palladium film, which were then analyzed at 10 kV using SEM.

### 2.18. In Vitro Release of Phenolic Compounds 

The produced MCs were subjected to phenolic compound release in vitro using the Minekus et al. [37] protocol with minor adjustments that are thoroughly explained in the Martinović et al. [38] paper.

### 2.19. In Vitro Simulated Digestion and Bioaccessibility Index

In vitro simulated digestion of the prepared MC was performed according to the INFOGEST protocol [39] with modifications. Initially, stock solutions representing conditions in the mouth (SSF—simulated salivary fluid), stomach (SGF—simulated gastric fluid), and intestines (SIF—simulated intestinal fluid) were prepared in accordance with Minekus et al. [37]. In vitro simulation of digestion was carried out in 5 test tubes, and each test tube represented a specific time interval (3, 63, 123, 183, and 243 min). A total of 0.1 g of MC was weighed into each test tube. After the addition of the simulated digestion solutions, the test tubes were placed on a vertical multipurpose rotator (PTR-60, Grant-bio Instruments, UK) placed in a thermostat (TC 135 S, Lovibond, Dortmund, Germany) that was preheated to 37 °C. After completion of certain phases, the test tube representing that phase was taken out of the rotator and centrifuged. The samples were centrifuged at 16,000× *g* and 4 °C for 30 min immediately after the test tubes were taken out of the rotator at the given time interval. After removing the supernatant, a 0.45 mm membrane filter (Syringe filters Spheros Nylon, Agilent Technologies, Inc., Santa Clara, CA, USA) was used. The following describes what was added at each stage of digestion:

Oral Phase (OP). A total of 4 mL of SSF + 25 μL of CaCl_2_(H_2_O)_2_ was added, and the pH was adjusted to 7 using 1 M HCl or 1 M NaOH (as appropriate). After that, redistilled water was added to a total volume of 10 mL.

Gastric Phase (GP). A total of 8 mL of SGF solution was added, along with 5 μL of CaCl_2_(H_2_O)_2_. The pH was adjusted to 3, and then 500 μL of pepsin was added, which was dissolved in redistilled water and then added so that its activity in the final solution was 2000 U/mL. Redistilled water was added to a total volume of 20 mL.

Intestinal Phase (IP). A total of 8.5 mL of SIF was added, along with 40 μL of CaCl_2_(H2O)_2_. The pH was adjusted to 7, then 5 ml of pancreatic solution (dissolved in the SIF solution so that its activity in the final volume was 100 U trypsin/mL) was added together with 2.5 mL of the bile extract solution (prepared in SIF so that the concentration of bile extract in the final solution was 1 mM). Redistilled water was added to a total volume of 40 mL.

Solid-phase extraction was used to remove impurities from the filtrate, which included salts, bile extract, and enzyme residues. This procedure was performed before the chromatographic analysis. A modified procedure, as described by Martinović et al. [38], was used for sample purification. 

The bioaccessibility index (BI) was calculated using Equation (14):

(14) $$BI\left(\%\right)=\left(\frac{C_{A}}{C_{B}}\right)\times 100$$

where *C*_A_ is the concentration of individual phenolic compounds after in vitro digestion and *C*_B_ represents the concentration of individual phenolic compounds before in vitro digestion.

### 2.20. Determination of Individual Phenolic Compounds

This study employed ultra-high-performance liquid chromatography (UHPLC Nexera XR, Shimadzu, Japan) using a photodiode detector to analyze the individual phenolic compounds in CSE in both qualitative and quantitative ways. A reversed-phase Kinetex^®^ C18 core-shell column (100 x 4.6 mm, 2.6 m, Phenomenex, Torrance, CA, USA) was used for the separation process. MC samples were prepared according to Tolun et al. [33] with minor modifications, in such a way that 0.1 g of MC was dissolved in 1.5 mL of a water/methanol/HCl mixture (89:10:1 *v*/*v*/*v*) and then centrifuged (Z 326 K, Hermle Labortechnik GmbH, Wehingen, Germany) at 14,000 rcf for 3 min. Prior to the UHPLC analysis, the supernatants were filtered through 0.45 μm membranes (Chromafil Xtra PTFE, Macherey-Nagel GmbH & Co. KG, Dueren, Germany). Software LabSolutions 5.87 was used to process the data. By comparing the retention durations and UV–vis spectra of the individual phenolic compounds with those of genuine standards that were examined under the same chromatographic conditions, the compounds were identified. The calibration curves created using the external standards were used for the quantification process. Individual phenolic compounds were determined using the UHPLC method described in Bucić-Kojić et al. [40].

### 2.21. Accelerated Stability Test 

The accelerated stability tests of the MCs, which proved their amorphous structure, were carried out using Binder KBF 240 equipment (Binder GmbH, Tuttlingen, Germany) with a constant climate chamber. Within the temperature range of 10 to 70 °C and RH of 10 to 80%, the temperature accuracy and reproducibility of the results were guaranteed using the electronically controlled APT.lineTM in-line preheating chamber and cooling system. A stability test was conducted at 40 ± 2 °C and 75 ± 2% RH in accordance with the paper by Cassol and Noreña [41]. Glass vials were used to store the samples for a period of three months. Sampling was performed after 0 days, 2 weeks, 1 month, and 3 months. 

### 2.22. Statistical Analysis

TIBCO Statistica software, Version 14.0.0.15 (TIBCO Software Inc., Palo Alto, CA, USA) was used to conduct a one-way analysis of variance (ANOVA) in order to determine the significance level of the difference between the arithmetic means of the samples that represented populations. Following an ANOVA that revealed statistically significant differences between the observed populations, a post hoc test (i.e., Duncan’s test for multiple ranges) was used to identify the populations between which a significant difference (*p* < 0.05) existed. In the figures or tables, the samples that belonged to the same population are identified by the same alphabetic letter.

## 3. Results and Discussion

### 3.1. Encapsulation Efficiency and Encapsulation Yield

A total of 17 encapsulation sets were carried out using GW as the main coating and selected carbohydrate co-coatings (T, S, MD, X) in varying proportions according to the experimental design described in Section 2.4. To determine the efficiency of the used coatings, the content of total phenolic compounds (TPC) and surface phenolic compounds (SPC) was determined using the prepared MCs as described in Section 2.5 and Section 2.6. The results are shown in Table 2.

The highest concentration of TPC (124.09 mg_GAE_/g_db_) was found for the MCs coated with GW (GW1), while for the other MCs coated with GW and co-coating, the concentration of TPC varied between 94.12 and 117.34 mg_GAE_/g_db_. The Folin–Ciocalteu method, a spectrophotometric method, is often used for a relatively rapid and inexpensive determination of TPC. However, it is known that the Folin–Ciocalteu reagent is not strictly specific for phenolic substances; it also reacted with other substances, including the proteins present in the samples. Since sample GW1 contained the highest amount of goat whey protein compared to all the other MCs, this could be the result of a slightly higher concentration of the measured TPC value, which does not necessarily correlate with the sum of the concentrations of all the quantified individual phenolic substances in the MCs. It can be observed that the increase in the amount of co-coating influences the decrease in the amount of TPC, regardless of the type of co-coating. On the other hand, the SPC values increased with the increase in the proportion of individual co-coatings and varied from 4.54 mg_GAE_/g_db_ (MD5) to 22.15 mg_GAE_/g_db_ (T30). In general, MD proved to be the co-coater that had the lowest concentration of non-encapsulated, i.e., surface phenolic compounds, and the MD samples differed least in their SPC content from the GW samples. A statistically significant difference in the content of TPC and SPC was recorded both within a particular group of co-coatings and in general between MCs.

**Figure 1:**
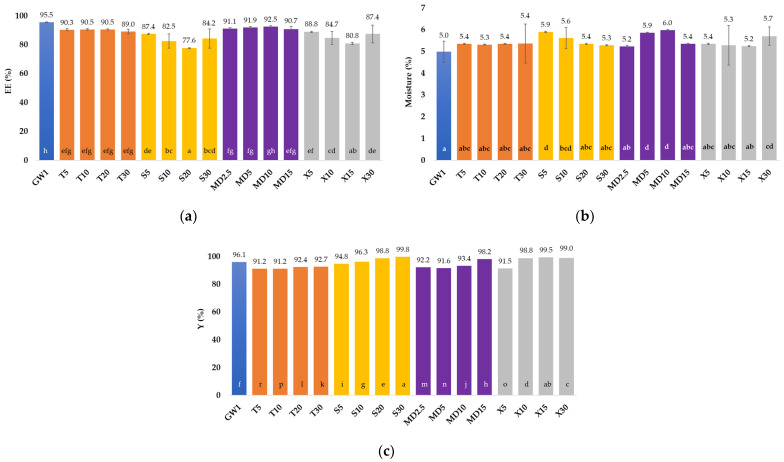
(**a**) Encapsulation efficiency (EE, %), (**b**) moisture content (%), and (**c**) encapsulation yield (Y, %) of microcapsules (MCs) prepared via spray draying with various coatings (coatings: GW (blue bar)—goat whey protein, T (orange bars)—trehalose, S (yellow bars)—sucrose, MD (purple bars)—maltodextrin (DE 4–7), X (gray bars)—xylose. The proportion of a single (co-)coating in relation to the total mass of the coating: 1–100%; 2.5–2.5%; 5–5%; 10–10%; 15–15%; 20–20%; 30–30%). Different letters (a, b, c…) represent statistically significant differences between the samples (ANOVA, post hoc Duncan test at *p* < 0.05).

The encapsulation efficiency (EE) of GPE with GW and GW in combination with four different co-coatings was evaluated based on the TPC and SPC values. The values for EE, the moisture content of the MCs, and the encapsulation yield (Y) were determined according to Section 2.7, Section 2.8 and Section 2.9, and the results obtained are shown in Figure 1.

When GW was used as a coating material for GPE spray drying, the EE reached 95.47% (Figure 1a). In the MCs encapsulated with a combination of GW and T coatings or MD, the amount of co-coating added had no effect on EE, whereas in the other two groups of MCs, the lowest EE was achieved with the application of 20% S and 15% X. In general, the inclusion of MD as a co-coating in conjunction with GW results in very high spray drying EE rates (91.1–92.5%). 

As seen in Figure 1b, the moisture content in the produced MC varied from 4.98% (GW1) to 5.98% (MD10), and visually, no agglomerate formation was noticed. The reduction in the total proportion of GW and the addition of the co-coatings resulted in an increase in the moisture in all the MC samples. A low moisture content, in the amount of 3–4%, was also reported by the authors Navarro-Flores et al. [42], who attributed this to the high drying inlet temperature that contributed to a higher rate of heat transfer to the particles, causing rapid water evaporation. A moisture level of less than 10% is thought to be adequate to ensure that the fruit powder produced via spray draying is microbiologically safe [43].

The Y value can be seen as an indicator of the value of a product for manufacturers. In encapsulation processes, Y can be defined as the output of the physical and chemical properties of MCs. With the help of the calculated Y values, it is possible to compare different encapsulation processes [44]. According to Tontul and Topuz [45], any spray drying with a Y value greater than 50% can be characterized as successful. The Y for all the encapsulations in this investigation was extremely high, higher than the 91.18% recorded for T5 (Figure 1c). We attributed the high Y to the use of GW protein as the main coating material, which improved the MC properties and minimized stickiness, ensuring a very high product yield (91.18–99.77%) in all the test series. Within the groups of MCs, it was observed that with the addition of a higher concentration of co-coating, the Y value also increased slightly. The samples with the addition of T had the lowest Y values on average.

### 3.2. Microcapsule Characteristics

**Figure 2:**
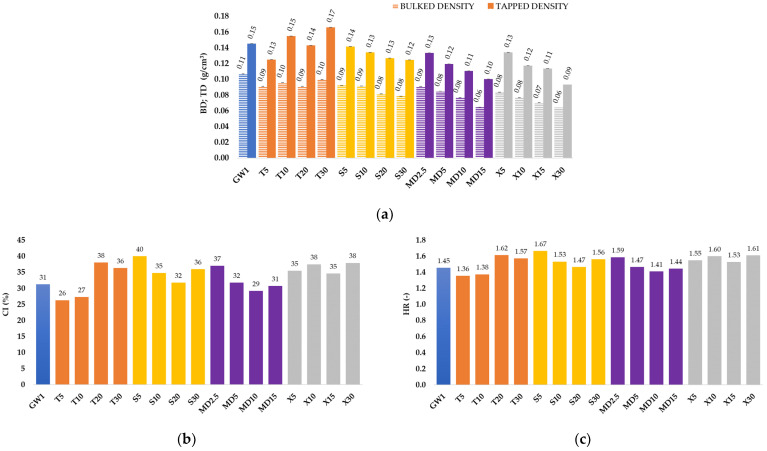
Density, flowability, and cohesiveness of spray-dried MCs: (**a**) bulk density (BD) and tap density (TD), (**b**) Carr index (CI), and (**c**) Hausner ratio (HR) of microcapsules (MCs) prepared via spray draying with various coatings (coatings: GW (blue bar)—goat whey protein, T (orange bars)—trehalose, S (yellow bars)—sucrose, MD (purple bars)—maltodextrin (DE 4–7), X (gray bars)—xylose. The proportion of a single (co-)coating in relation to the total mass of the coating: 1–100%; 2.5–2.5%; 5–5%; 10–10%; 15–15%; 20–20%; 30–30%).

Density (bulk density, BD and tap density, TD), flowability, and cohesiveness are important properties of powders. The high BD of MCs is favorable for lowering transportation and packaging costs [45]. Lower product BDs are undesirable, since they necessitate more container space. Furthermore, as the bulk density decreases, more air is trapped inside the MCs, and the product is more likely to oxidize, resulting in decreased storage stability [46]. The specified properties of the MCs in this paper were determined as stated in Section 2.10 and Section 2.11, and the results are shown in Figure 2. 

All the experiments showed low density values (Figure 2a), which, according to Braga et al. [47], may be a consequence of the high inlet temperature. Within the S, X, and MD groups, the value of BD and TD decreased with increasing concentrations of the added co-coatings. According to the classification of the European Pharmacopoeia [48] and the calculated values for CI (Figure 2b) and HR (Figure 2c), the flowability and cohesion of the MC for the GW1, T5, T10, MD10, and MD15 samples can be characterized as poor, with a CI between 26–31% and HR of 1.35–1.45. Samples T20, S5, X10, and X30 had a very poor flowability (CI = 32–37%, HR = 1.46–1.59), and all the other samples have a very poor fluidity (CI > 38%, HR > 1.60). The poor flowability of all the produced MCs can be attributed to the large proportion of whey proteins, which have a significant proportion of fat in their composition and are therefore exhibit poor flowability. The size of the MCs also has an effect on the flowability of the MCs. As the particle size decreases, the specific surface area increases, and this leads to reduced flowability. A larger contact area becomes available, especially for cohesive forces, resisting flow frictional forces. In addition, the reduced flowability of the MCs can also be caused by an increase in humidity, as this results in an increase in the effects of the capillary forces between the MC particles [49]. Furthermore, according to Tontul and Topuz [45], the surface of the particles affects the bulk density of the MCs, i.e., the smoother and more uniform the particles are, the higher the bulk density value.

Particle size, shape, and size distribution also play essential roles in MC food processing, handling, and shelf life, with particle microstructure influencing various MC qualities such as stability and fluidity. The drying technique and parameters have a significant impact on particle size, shape, and distribution [50]. Table 3 shows that the average particle size of the produced MCs varied from 3.31 μm (S30) to 4.61 μm (MD2.5). However, within the MD group of MCs, it was noticed that with an increase in the concentration of added MD from 2.5 to 10%, the particle size decreased. According to Medina-Torres et al. [51], a particle qualifies as fine if its average diameter is less than 5 μm. Accordingly, every coating material combination we have used produces a fine powder. After contrasting our findings with those of other authors, we came to the conclusion that our particles were more uniform and smaller than those of other spray-dried fruits with carriers. The average particle size of acai MCs ranged from 9.33 μm to 13.67 μm [52], while the particle size of ripe mango fruit MCs was 88.879 μm [53], whereas the particle size of baobab fruit MCs was 953 μm [54]. During the production of whey protein isolate particles with trehalose used as a stabilizer, the particle size varied from 100 to 1000 nm [29]. Nonetheless, the small particle size of our samples is consistent with the claims made by Verruck et al. [28] and Ćujić-Nikolić et al. [55]. Table 3 shows that sample GW1 had the highest span value (2.20), which means that it was the least homogeneous sample, i.e., the one with the widest particle size distribution, indicating the uniformity of the sample. As a result, it also had the highest bulk density of 0.11 g/cm^3^. Sample X30 was the most homogenous sample, i.e., the sample with the smallest span value of 1.73. In general, the entire group of samples with X used as the co-coating had the lowest span values of 1.73–1.97.

The solubility parameters WSI, WAI, and SP are important indicators of the functional properties of the product and are indicators in the application and storage of MCs. WSI represents the ability of the MCs to dissolve in water. The desirability of a higher WSI value depends on the final application of the MC product. If the MC is intended to be used in the food or pharmaceutical industries, a high WSI is especially important so that the MCs are easily incorporated and evenly distributed within the final product. WAI values are related to the degree of gelatinization and microbial stability. The higher the WAI values, the greater the possibility of microbiological instability of the MC products [19]. The solubility properties of the MCs determined in this study are listed in Table 4. 

The MCs with X used as the co-coating showed the highest water solubility (WSI). Within that group, the WSI values varied from 51.80 to 63.15%. In addition, the MCs from the group showed the highest WAI values (4.60–5.18) as well as the highest SP values (10.75–13.93). The small size and uniformity of the capsules certainly contributed to the good solubility of the X microcapsules. In contrast to X, the use of MD as the co-coating material resulted in MCs with the lowest WSI and SP values. Within that group, the WSI ranged from 43.91 to 51.17% and the SP values ranged from 8.11 to 9.64. The reason for this may be cross-linking of MD with other compounds, which results in lower values of solubility and swelling ability compared to the other samples. During encapsulation via spray drying the phenolic mango extract, MCs with lower WSI (8.62% to 24.28%) and WAI (2.58% to 3.91%) values were obtained [19]. Unlike these MCs, Sidlagatta et al. [56] reported a WSI value of 77.9 to 89.8% as well as WAI values of 6.7 to 12.3% for a spray-dried sweet orange MC.

Based on the value of the contact angle of water and diiodomethane, the polarity of the samples was calculated as described in Section 2.14, which ranged from 36.46% (MD2.5) to 48.39% (S20), as shown in Table 4. It can be concluded that all the prepared MCs are hydrophilic, which indicates their good solubility in water. According to Chang et al. [57], many spray-dried plant extracts contain a considerable amount of hydrophilic substances in their composition, which makes them hygroscopic. 

**Figure 3:**
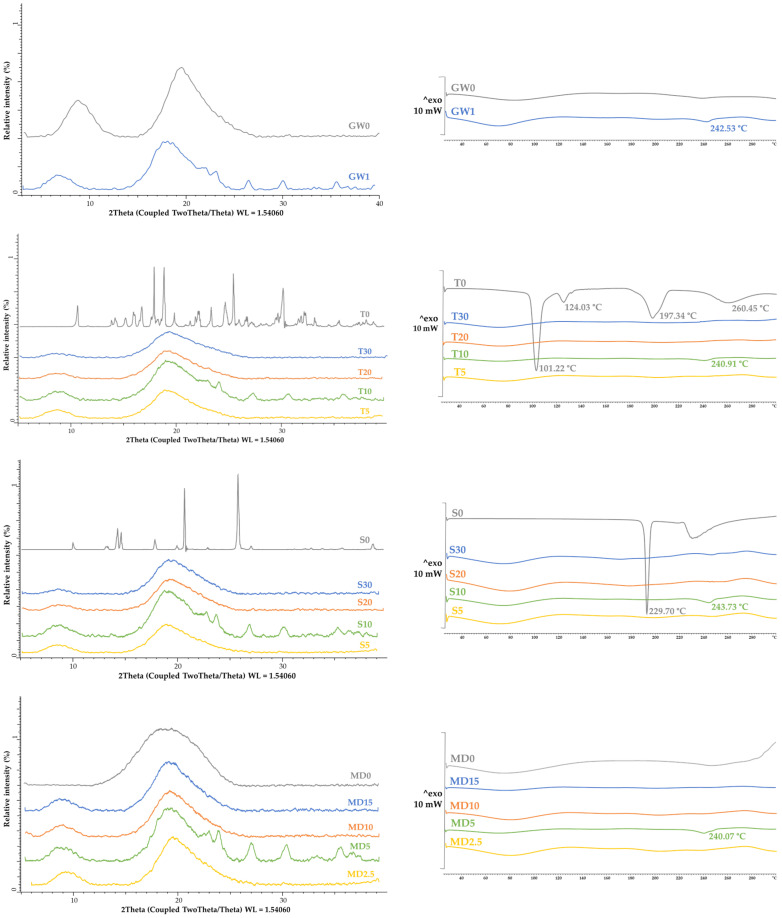

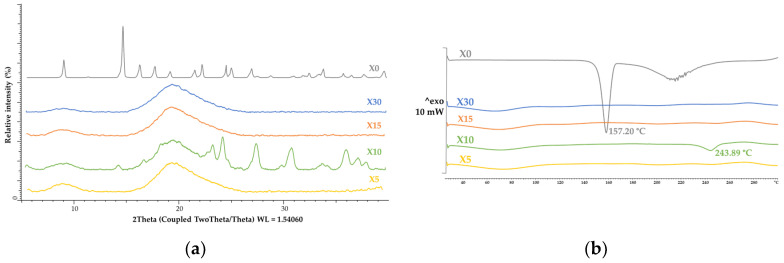
Crystallinity and amorphousness of MC samples with various coatings: (**a**) X-ray powder diffractograms and (**b**) DSC thermograms.

X-ray powder diffraction (XRPD) and differential scanning calorimetry (DSC) analyses were used to investigate the crystallinity and amorphousness of the MC samples. The XRPD patterns and the DSC thermograms of the MC samples are shown in Figure 3. As stated by numerous authors, spray drying produces mostly amorphous MCs. Figure 3a shows that the co-coating materials S (S0), T (T0), and X (X0) have a crystalline structure. The spray-dried MCs containing 10% T (T10), S (S10), X (X10), 5% MD (MD5), and MC containing only GW (GW1) had a partially crystalline structure, while all the other MCs had an amorphous structure according to the XRPD results, as did the GW (GW0) and MD (MD0) coatings. Spray drying of fruit juices, according to Cano-Chauca et al. [58], results in MCs with a high proportion of sugars present in an amorphous state due to the drying process used. 

Because these sugars are exceedingly hygroscopic, they can crystallize by absorbing only a small amount of water. Because the ordered system of the crystalline structure promotes product stability, this type of structure in final products is constantly sought. By investigating the effects of various conditions on water-induced crystallization across different amorphous materials obtained via spray drying, the authors Chiou and Langrish [59] came to similar conclusions. They verified that products require longer times to crystallize when their molecular weight and glass transition temperature are higher. Since grape pomace extract is also rich in numerous sugars, it is possible that water adsorption is precisely the reason for the partially crystalline structure of the mentioned samples. During the crystallinity test, in contrast to our research, the addition of sugar by the authors Haque et al. [29] did not cause the appearance of peaks on the X-ray diffractograms, and thus the structures of these MCs were characterized as amorphous. The use of cellulose or waxy starch in combination with MD caused a partially crystalline structure of the soy sauce MCs [60].

The DSC thermograms (Figure 3b) of MC samples GW1, T10, S10, MD5, and X10 showed endothermic peaks at a temperature of around 240 °C, which confirms the partially crystalline structure proven by the XRPD analysis. Slightly broadened peaks are also visible from the DSC curves of the spray-dried MC samples, indicating water loss. Endothermic peaks are visible in the DSC curves of the samples of pure co-coatings with crystalline structures (T0, S0, and X0), indicating their melting points.

**Figure 4:**
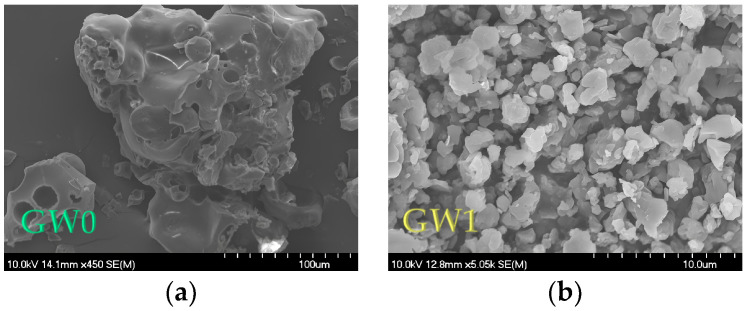
Scanning electron micrographs for: (**a**) goat whey protein particles (GW0), (**b**) MCs coated with GW (GW1).

**Figure 5:**
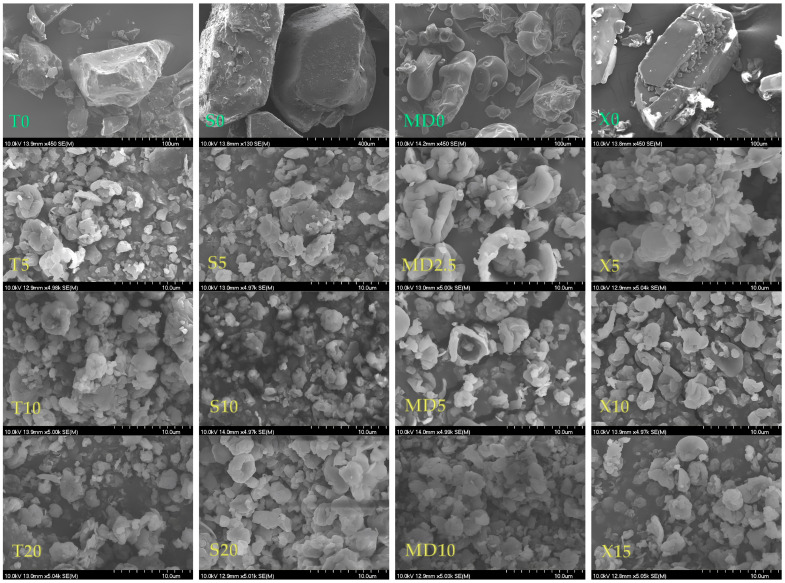

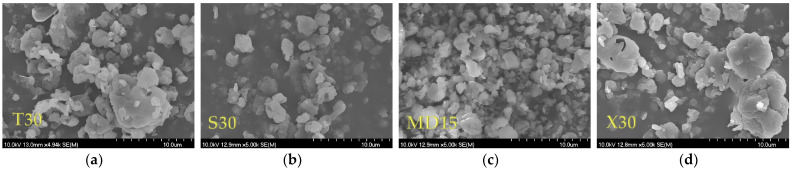
Scanning electron micrographs for: (**a**) trehalose particles (T0) and MCs coated with a combination of GW and T (T5, T10, T20, T30); (**b**) sucrose particles and MCs coated with a combination of GW and S (S5, S10, S20, S30); (**c**) maltodextrin (DE 4–7) particles and MCs coated with a combination of GW and MD (MD2.5, MD5, MD10, MD15); (**d**) xylose particles (X0) and MCs coated with a combination of GW and X (X5, X10, X15, X30).

The scanning electron microscope (SEM) images of the coatings and MC samples are shown in Figure 4 (GW and MCs coated with GW) and Figure 5 (T, S, MD, X, and MCs coated with GW and co-coating in different ratios). It can be seen that all the MC particles had an approximately spherical shape, their surface was not smooth, and the particles were not uniform in size and morphology. This is one of the explanations for the low density of our MCs, and it confirms the results of the size distribution parameters (Table 3).

It can be clearly seen (Figure 4 and Figure 5) that the coating particles (GW0, T0, S0, MD, and X0) were larger than the MCs produced via spray drying. The MD0 particles were closer to the spherical form, while the rest of the coatings were irregular particles. Also, the hollow structure of the GW itself was very noticeable, which was not visible in the spray-dried, GW-coated MCs (Figure 4a).

In all the SEM micrographs shown in Figure 5, it is visible that the combination of GW coatings with co-coatings created MCs whose particles were of different, irregular shapes and sizes, with some of them being in agglomerates. The size and surface appearance of each group of MCs created via spray drying were observed to differ significantly from the coating and co-coating materials. The shriveling of the MCs is explained as a consequence of fast water evaporation due to spray drying. Navarro-Flores et al. [42] stated that the rapid evaporation of water can result in the hardening of the capsule, which leads to retention of the original shape or the evaporation of water and the formation of shriveled microcapsules.

According to Haque et al. [29], the wrinkles on the surface of whey protein MCs are the result of the formation of a “skin”, which is characteristic of all protein-rich substances. This property, combined with the rapid evaporation of water during spray drying, results in particles that exhibit protrusions. These deviations from the regular spherical shape of all MCs are the result of the poor flow properties reflected in the CI and HR values (Figure 2) and good solubility properties (Table 4). According to Oliveira et al. [61], such protrusions can have a negative influence on the flow properties of the MCs, but they have no influence on the stability of the MCs. It is also noted that the addition of sugar as a protein stabilizer of GW has an influence on the morphology of the particles, i.e., the MCs with the addition of T, S, and X had smoother surfaces compared to the GW1 MCs. In the case of MCs with the addition of X, the smooth pits were most pronounced. A hollow structure can also be seen in the image for the MD particles. In their study, the authors Wijiani et al. [62] reported the same effect of the addition of S on the morphology of the particles. In this study, the particles with added S had a smoother surface than the particles without sucrose in their composition.

Based on the results of the above analyses, one representative with good properties (GW1, T10, S10, MD5, and X10) was selected from each MC group to perform in vitro release and in vitro digestion simulation experiments.

### 3.3. In Vitro Release of Phenolic Compounds from MCs

The encapsulation technique allows the matrix to remain isolated from the external environment and offers stability in unfavorable circumstances until the compound needs to be released. Matrix release can be caused by a variety of factors, some of which include diffusion, degradation, solvents, pH, temperature, and pressure [63]. The release of phenolic compounds from the spray-dried MCs took place in the oral, gastric, and intestinal phases using solutions without enzymes that simulate the conditions in a particular phase of the gastrointestinal tract. The use of the coating material enables the protection of phenolic compounds from adverse environmental conditions such as low pH values in the gastric phase and ensures safe transport and release in the intestinal phase [38]. Phenolic compounds are adsorbed in the intestinal phase; about 46% in the small intestine and 42% in the large intestine [64]. The adsorption of phenolic compounds in the intestinal phase enables the manifestation of their numerous biological activities, such as antioxidant, antimicrobial, or antiproliferative activities [65].

**Figure 6:**
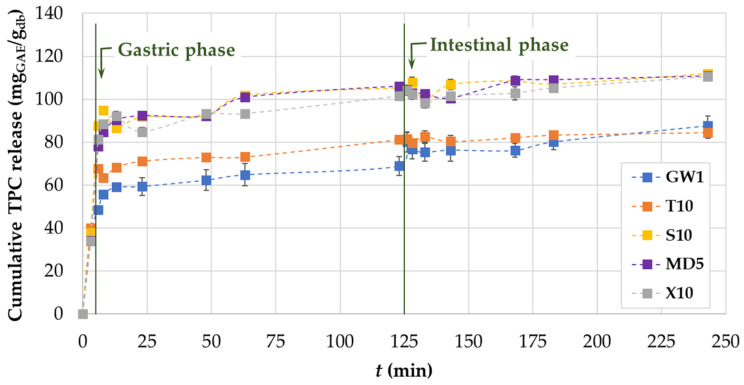
Cumulative release of TPC (mg_GAE_/g_db_) from selected spray-dried MCs (GW1, T10, S10, MD5, and X10) in three digestive phases.

The in vitro release of phenolic compounds test was performed according to the protocol described in Section 2.18. The results are shown in Figure 6 as the cumulative release of TPC from the MCs.

The cumulative release rate in the oral phase ranged from 34.13% (MD5) to 40.03% (T10) of the total TPC released at the end of the test (Figure 6). The diffusion of phenolic compounds then continued in the gastric phase. During the release in the gastric phase due to the pH change to acidic conditions, the color of the electrolyte solution containing the MC changed to red–pink, which was due to the presence of anthocyanins in the grape pomace extract. The cumulative release rate in the gastric phase ranged from 48.56% (GW1) to 100.84% (MD5). Within the last phase of the simulated gastrointestinal digestion, the values of the cumulative released TPC ranged from 76.10% (GW1) to 111.92% (S10). It is important to note that samples X10 and MD5 also had high values of released cumulative TPC in the amount of 110.42 and 110.75%, respectively. Our findings, which show a higher release of phenolic compounds during the intestinal phase of digestion as opposed to the gastric phase, are consistent with the results reported by Dag et al. [66], who evaluated the release of phenolic compounds from freeze-dried goldenberry juice MCs. Recent studies on delivery system engineering show that protein–polysaccharide blend systems hold great promise for improving the release, retention, and protection of bioactive compounds [67]. As stated by Belščak-Cvitanović et al. [68], the hydrophilic character of the used polysaccharide and protein coatings as well as their water sorption properties cause high and rapid release of bioactive compounds in digestive fluids. The strong resistance of β-lactoglobulin to pepsin [69] is another factor in favor of this. When using these combinations of coating materials, the high value of TPC cumulative release could be attributed to all of the aforementioned factors. The values of TPC released from the MCs in which T was used as a co-coating were lower compared to all the other co-coatings and were closer to the values of TPC released from the MCs in which only GW was used as a coating (GW1). A potential reason for this reduced phenolic release may be that T is less soluble in simulated gastric and intestinal fluids than the rest of the co-coating materials, which makes T-MC less effective in releasing phenolic compounds. 

### 3.4. In vitro Simulated Digestion

In vitro simulated digestion is an accepted approach for estimating target molecule bioaccessibility. It can provide further information regarding the metabolism of phenolic compounds, their availability for further absorption in the body, and their potential health benefits. Phenolic compounds must be liberated from the microparticle matrix during gastrointestinal digestion in order to become bioavailable. Therefore, in this study, the influence of different coatings on the bioaccessibility index (BI) of individual phenolic components from GPE was investigated using in vitro simulated digestion testing according to the protocol described in Section 2.19 for 243 min, comprising 3 digestion phases: 3 min in the oral phase (OP), 120 min in the gastric phase (GP), and a further 120 min in the intestinal phase (IP). The content of individual phenolic substances was determined before and after a certain period of digestion, and the results of the UHPLC analysis (according to Section 2.20) are listed in Table 5 and Table 6. Before digestion, a total of 21 individual phenolic components were quantified in the dissolved microcapsules. After 243 min of simulated digestion in vitro, 10 of the previously quantified phenolic components were not detected in the digestate of any type of MC, namely: procyanidin B1, caffeic acid, chlogenic acid, syringic acid, *p*-coumaric acid, ellagic acid, rutin, resveratrol, kaempferol, and quercetin (Table 5). 

The remaining 11 phenolic compounds quantified in the MCs prior to digestion were also quantified in the digestion product after 243 min of simulated digestion in vitro (Table 6).

**Figure 7:**
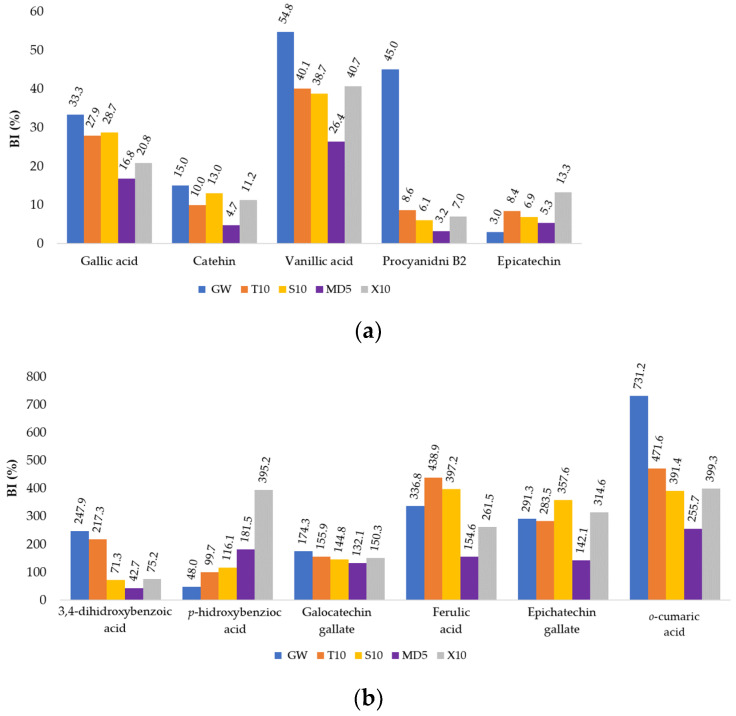
Bioaccessibility index (BI, %) of the individual phenolic substances of MCs depending on the coatings used: (**a**) BI < 100%, (**b**) BI > 100%.

After digestion, the contents of five of the phenolic compounds decreased: gallic acid, catechin, vanillic acid, procyanidin B2, and epicatechin, compared to the amount in the MCs before digestion, resulting in a BI of <55% (Figure 7a). It is possible that these compounds were hydrolyzed by intestinal enzymes and therefore their content was reduced after digestion [70]. However, it is important to point out that gallic acid, 3,4-dihydrohibenzoic acid, catechin, vanillic acid, and ferulic acid as well as epicatechin gallate were detected during the intestinal digestion phase, while they were not present in the oral or gastric digestion phase (Table 6). High concentrations of individual compounds in the digestate after the intestinal digestion phase were found for 3,4-dihydrohibenzoic acid, *p*-hydroxybenzoic acid, galocatechin gallate, and *o*-coumaric acid. More specifically, their higher concentrations were quantified in the digestate compared to in the MCs of the same type before digestion, resulting in a BI > 100% (Figure 7b). It can be assumed that the increased concentration of the compounds mentioned is due to the breakdown of more complex compounds during digestion, such as various anthocyanins, which are most abundant in the gastric phase of digestion, i.e., at acidic pH values. 3,4-dihydroxybenzoic acid can be a degradation product of oenin chloride and also of curomannin chloride. It is to be expected that this is also the case for the other phenolic acids mentioned. The high BI value of gallocatechin gallate could be due to the hydrolysis of highly polymerized compounds such as procyanidin B1, which in this case was not quantified in the samples at the end of digestion.

In the case of gallic acid, the BI values varied from 16.79% (MD5) to 33.32% (GW1) (Figure 7a). Conversely, in the case of o-coumaric acid, the BI values ranged from 255.67% (MD5) to 731.23% (GW1), and these were the highest values of BI in this experiment (Figure 7b). 

### 3.5. Accelerated Stability of MCs 

Amorphous materials are thermodynamically in a non-equilibrium state and therefore tend to convert to crystalline (thermodynamically stable) structures during storage. The rate of this transformation depends primarily on temperature and relative humidity (RH) [17]. When stored in a high RH environment, they absorb moisture and subsequently recrystallize [71].

**Figure 8:**
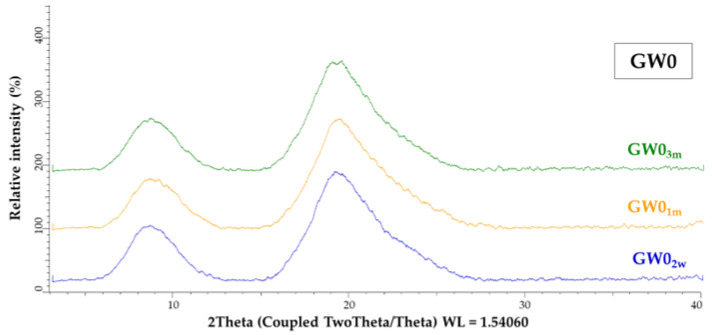
XRPD pattern of GW powder (GW0) after accelerated ageing for 2 weeks (GW1_2w_), 1 month, (GW1_1m_) and 3 months (GW1_3m_).

**Figure 9:**
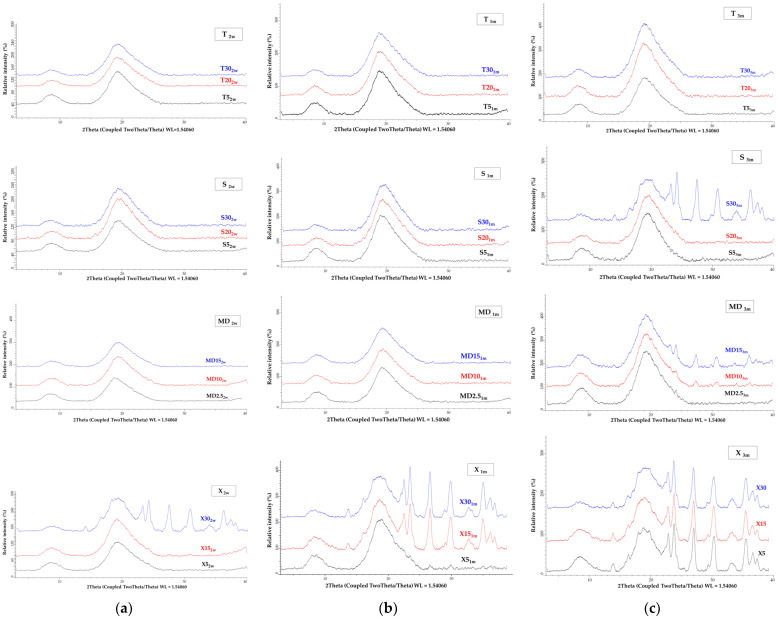
XRPD pattern of MCs coated with a combination of GW and T, S, MD, and X after accelerated ageing for (**a**) 2 weeks, (**b**) 1 month, and (**c**) 3 months.

To check the stability of the powders, samples of the pure protein coating (GW0) and samples of microcapsules whose X-ray powder diffractograms (Figure 3) indicated an amorphous structure (T5, T20, T30, S5, S20, S30, MD2.5, MD10, MD15, X5, X15, and X30) were subjected to the accelerated ageing test as described in Section 2.21. The X-ray powder diffractograms of the samples after the accelerated ageing test over 2 weeks, 1 month, and 3 months are shown in Figure 8 and Figure 9. 

The amorphous structure and stability of the GW powder was maintained even after three months of accelerated aging (Figure 8). After a two-week acceleration stability test (Figure 9a), only the structure of sample X30 (X30_2w_) changed from an amorphous to a partially crystalline structure. All the other samples remained amorphous. The MC samples with the xylose co-coating, X5 and X15 (X5_1m_ and X15_1m_), also changed their structure from amorphous to semi-crystalline after one month of accelerated ageing (Figure 9b). After three months, sample S30 (S30_3m_) and two samples with MD addition (MD15_3m_ and MD10_3m_) showed a change in their structure from an amorphous to a semi-crystalline form. At the same time, samples S5_3m_, S20_3m_, T5_3m_, T20_3m_, T30_3m_, and MD2.5_3m_ proved their stability, as no structural changes were detected even after three months under the conditions of the accelerated stability test (Figure 9c).

**Figure 10:**
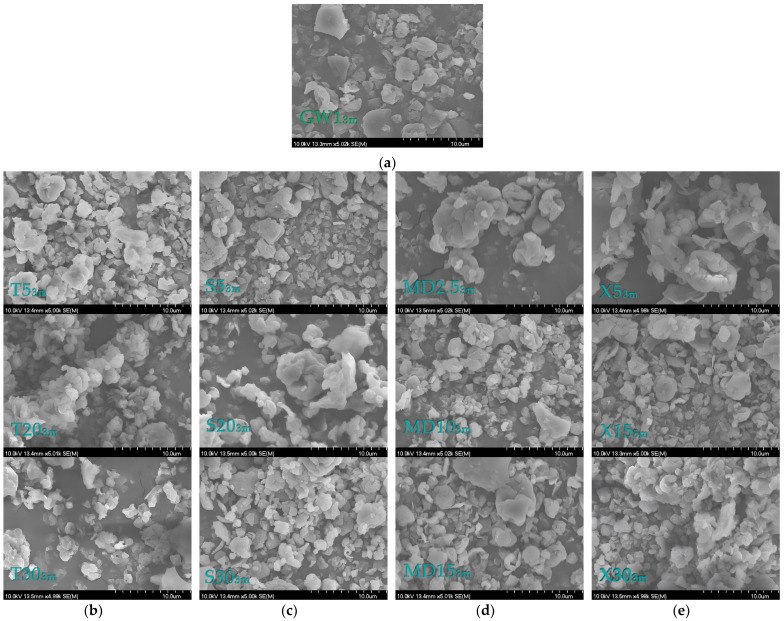
Scanning electron micrographs for powders/MCs after 3 months of the accelerated stability test: (**a**) GW0_3m_; (**b**) T5_3m_, T20_3m_, T30_3m_; (**c**) S5_3m_, S20_3m_, S30_3m_; (**d**) MD2.5_3m_, MD10_3m_, MD15_3m_; and (**e**) X5_3m_, X15_3m_, X30_3m_.

In addition, SEM analyses of the MC samples were made after three months under the conditions of the accelerated stability test. For the majority of the samples, no appreciable alterations in the microcapsule’s appearance were noticed (Figure 10). For sample X15_3m_, there was an apparent cavity. We can infer that trehalose addition, even in the smallest amounts, is necessary to stabilize the main coating material because no changes in the crystallographic structure of the particles were observed in samples T5_3m_, T20_3m_, T30_3m_. Furthermore, the same results were seen in the samples (MD2.5_3m_) that had the least amount of MD added.

## 4. Conclusions

Spray drying of grape pomace extract with GW and the addition of T, X, S, or MD as a protein stabilizer produces MCs with high Y values (91.49–99.77%) with a small proportion of surface phenolic compounds (4.54–22.15 mg_GAE_/g_db_). Analysis of the MCs revealed that they were hydrophilic MCs with a mostly amorphous structure, which was determined via XRPD and DSC analysis. The MCs with the addition of 10% xylose, trehalose, or sucrose and the MCs with the addition of 5% MD had a partially crystalline structure that could be attributed to naturally occurring sugars in a sample of grape pomace extract and their water-induced crystallization. The average size of the particles varied from 3.31 to 4.61 μm, and the recorded span values were low, which suggests the uniformity of the particles. The bulk and tapped densities were are low compared to the results of other authors, and the flowability of the MCs was characterized as poor or very poor. The high spray drying temperature can be the cause of the MCs’ low density, as well as the high proportion of protein coating material whose fluidity is compromised by the amount of fat in its composition, causing its poor fluidity. Additionally, the specific surface area has an impact on fluidity; the smaller the particle, the larger the contact area that becomes available, particularly for cohesive forces and frictional forces that resist flow. By conducting an in vitro release assay, the values of cumulative released TPC increased from phase to phase, reaching values as high as 111.92% in the intestinal phase. In comparison to the MCs containing MD, X, and S, the MCs containing T achieved lower values of cumulatively released TPC. Performing in vitro simulated digestion had a positive effect on the bioavailability of specific individual phenolic compounds. The bioavailability index for *o*-coumaric acid varied between 255.67% and 731.23% depending on the coating used. The use of T as a co-coating material resulted in MCs with an exceptional stability, even after 3 months. Conversely, the final MCs were less stable when X was used as a coating material in any of the tested concentrations.

The results of this research show that the use of GW as the main coating material resulted in highly successful encapsulation processes and high-quality MCs with the capacity to maintain and enhance the bioaccessibility of phenolic compounds during in vitro simulated digestion. All of the above evidence points to the possibility of using GW as a coating material using the spray drying process, with the end product finding applications in the food, pharmaceutical, and nutraceutical industries.

## Figures and Tables

**Table 1:** Percentage ratio of coatings and co-coatings in various encapsulation sets of encapsulations of GPE.

Sample Label	Coating	(%)	Co-Coating	(%)
GW1	goat whey protein	100	-	-
T5	goat whey protein	95	trehalose	5
T10	goat whey protein	90	trehalose	10
T20	goat whey protein	80	trehalose	20
T30	goat whey protein	70	trehalose	30
S5	goat whey protein	95	sucrose	5
S10	goat whey protein	95	sucrose	10
S20	goat whey protein	90	sucrose	20
S30	goat whey protein	80	sucrose	30
MD2.5	goat whey protein	97.5	maltodextrin (DE 4–7)	2.5
MD5	goat whey protein	95	maltodextrin (DE 4–7)	5
MD10	goat whey protein	90	maltodextrin (DE 4–7)	10
MD15	goat whey protein	85	maltodextrin (DE 4–7)	15
X5	goat whey protein	95	xylose	5
X10	goat whey protein	90	xylose	10
X15	goat whey protein	85	xylose	15
X30	goat whey protein	70	xylose	30

**Table 2:** Total phenolic content (TPC) and surface phenolic content (SPC) of microcapsules (MCs) prepared via spray draying with various coating (s).

Sample ^1^	TPC (mg_GAE_/g_db_) ^2^	SPC (mg_GAE_/g_db_) ^2^
GW1	124.09 ± 1.63 ^i^	5.62 ± 0.34 ^bc^
T5	102.63 ± 1.19 ^e^	9.9 ± 0.61 ^f^
T10	114.37 ± 0.68 ^g^	10.89 ± 0.54 ^g^
T20	113.15 ± 0.66 ^g^	14.21 ± 0.52 ^i^
T30	98.84 ± 0.85 ^d^	22.15 ± 0.15 ^l^
S5	101.47 ± 1.52 ^e^	8.98 ± 0.53 ^e^
S10	102.63 ± 0.85 ^e^	7.70 ± 0.62 ^d^
S20	97.50 ± 0.81^cd^	10.92 ± 0.27 ^g^
S30	86.81 ± 1.54 ^a^	16.66 ± 0.67 ^k^
MD2.5	113.12 ± 1.18 ^g^	7.12 ± 0,20 ^d^
MD5	97.38 ± 0.82 ^c^	4.54 ± 0.27 ^a^
MD10	96.89 ± 1.33 ^c^	5.05 ± 0.59 ^ab^
MD15	93.30 ± 1.38 ^b^	5.71 ± 0.26 ^c^
X5	96.78 ± 1.60 ^c^	8.93 ± 0.49 ^e^
X10	117.34 ± 1.28 ^h^	9.89 ± 0.39 ^f^
X15	104.10 ± 0.95 ^f^	12.89 ± 0.46 ^h^
X30	94.12 ± 1.56 ^b^	15.78 ± 0.34 ^j^

^1^ Coatings: GW—goat whey protein, T—trehalose, S—sucrose, X—xylose, MD—maltodextrin (DE 4–7); The proportion of a single (co-)coating in relation to the total mass of the coating: 1–100%; 2.5–2.5%; 5–5%; 10–10%; 15–15%; 20–20%; 30–30%. ^2^ All data are expressed as the mean value of replication ± SD. Different letters (a, b, c…) represent statistically significant differences between the samples (ANOVA, post hoc Duncan test at *p* < 0.05).

**Table 3:** Size distribution parameters of MCs.

Sample	Mean Diameter, d (0.5) (μm)	Specific Surface Area (m^2^/g)	Span (-)
GW1	3.76	2.00	2.20
T5	3.78	2.04	2.09
T10	3.36	2.22	2.18
T20	3.61	2.07	2.08
T30	3.38	2.19	2.03
S5	3.76	2.01	1.93
S10	3.50	2.13	2.12
S20	3.51	2.12	1.90
S30	3.31	2.21	1.80
MD2.5	4.61	1.68	2.05
MD5	3.88	1.97	2.02
MD10	3.37	2.21	1.99
MD15	3.45	2.16	2.16
X5	3.53	2.11	1.97
X10	3.88	1.94	1.84
X15	3.32	2.21	1.94
X30	3.81	1.93	1.73

**Table 4:** Solubility properties of MCs: water solubility index (WSI), water adsorption index (WAI), swelling capacity (SP), and polarity.

Sample	WSI (%)	WAI (-)	SP (-)	Polarity (%)
GW1	57.47 ± 0.54 ^f^	4.37 ± 0.01 ^ab^	10.28 ± 0.10 ^fghi^	46.47 ± 0.32
T5	51.40 ± 0.97 ^cd^	4.82 ± 0.24 ^efg^	9.92 ± 0.28 ^defg^	43.93 ± 2.71
T10	55.25 ± 0.26 ^e^	4.68 ± 0.01^cde^	10.45 ± 0.03 ^ghi^	45.99 ± 0.89
T20	56.46 ± 0.69 ^ef^	4.67 ± 0.13 ^cde^	10.74 ± 0.14 ^hi^	44.80 ± 1.23
T30	55.63 ± 0.22 ^e^	4.24 ± 0.07 ^a^	9.56 ± 0.10 ^cdef^	44.78 ± 1.17
S5	50.78 ± 0.80 ^bcd^	4.64 ± 0.37 ^bcde^	9.44 ± 0.90 ^bcde^	40.06 ± 1.19
S10	50.83 ± 1.46 ^bcd^	4.96 ± 0.44 ^fgh^	10.07 ± 0.59 ^efgh^	44.01 ± 0.36
S20	49.55 ± 1.36 ^b^	4.62 ± 0.01 ^bcde^	9.15 ± 0.23 ^bc^	48.39 ± 1.25
S30	57.68 ± 2.48 ^f^	4.57 ± 0.13 ^bcde^	10.80 ± 0.33 ^i^	48.25 ± 0.93
MD2.5	49.63 ± 2.21^bc^	4.41 ± 0.04 ^abc^	8.77 ± 0.46 ^ab^	36.46 ± 1.74
MD5	51.17 ± 1.86 ^bcd^	4.7 ± 0.04 ^def^	9.64 ± 0.46 ^cdef^	47.82 ± 1.62
MD10	50.79 ± 0.15 ^bcd^	4.55 ± 0.12 ^bcde^	9.25 ± 0.28 ^bcd^	41.50 ± 1.72
MD15	43.91 ± 0.21 ^a^	4.55 ± 0.04 ^bcd^	8.11 ± 0.04 ^a^	41.24 ± 0.28
X5	51.80 ± 0.19 ^d^	5.18 ± 0.63 ^h^	10.75 ± 1.27 ^hi^	40.65 ± 0.18
X10	62.62 ± 4.20 ^g^	5.08 ± 0.00 ^gh^	13.68 ± 1.52 ^k^	43.85 ± 0.51
X15	62.59 ± 0.40 ^g^	4.60 ± 0.13 ^bcde^	12.31 ± 0.49 ^j^	43.42 ± 0.63
X30	63.15 ± 0.07 ^g^	5.13 ± 0.22 ^h^	13.93 ± 0.58 ^k^	47.42 ± 2.36

All the data are expressed as the mean value of the replications ± SD. Different letters (a, b, c…) represent statistically significant differences between the samples (ANOVA, post hoc Duncan test at *p* < 0.05).

**Table 5:** Concentration of individual phenolic compounds quantified in MCs before digestion but not after 243 min of in vitro simulated digestion.

Phenols (μg/g_db_)	Samples				
	GW1	T10	S10	MD5	X10
Procyanidin B1	1121.04 ± 48.47	1054.90 ± 26.64	923.51 ± 40.55	1329.88 ± 61.03	987.87 ± 90.96
Caffeic acid	23.97 ± 0.28	21.89 ± 0.13	19.78 ± 0.29	21.49 ± 0.25	17.21 ± 0.50
Chlorogenic acid	81.32 ± 1.73	67.99 ± 4.58	77.79 ± 1.30	89.30 ± 2.33	70.14 ± 1.01
Syringic acid	122.55 ± 1.83	204.17 ± 4.07	182.78 ± 0.80	199.24 ± 1.55	158.92 ± 1.30
*p*-coumaric acid	11.55 ± 0.08	10.61 ± 0.17	9.82 ± 0.09	14.13 ± 0.11	6.61 ± 0.59
Ellagic acid	84.07 ± 0.86	100.32 ± 2.25	90.88 ± 2.30	87.67 ± 2.97	83.06 ± 1.90
Rutin	159.54 ± 2.11	189.07 ± 5.20	152.52 ± 0.73	196.83 ± 0.92	218.63 ± 9.55
Resveratrol	19.93 ± 0.03	26.89 ± 0.49	23.54 ± 0.29	26.02 ± 0.70	20.26 ± 1.38
Kaempferol	21.13 ± 0.55	20.98 ± 0.20	23.69 ± 0.42	25.44 ± 0.15	20.21 ± 0.38
Quercetin	317.88 ± 2.76	395.91 ± 0.58	351.92 ± 4.97	359.10 ± 0.23	303.78 ± 2.72

Phenolic contents are expressed as mean value (μg/g_db_) ± SD.

**Table 6:** Concentration of individual phenolic compounds quantified in MCs before digestion and during different stages of in vitro simulated digestion.

Phenols/Sample	Before Digestion	Oral Phase	Gastric Phase		Intestinal Phase	
		OP_3_	GP_63_	GP_123_	IP_183_	IP_243_
Gallic acid (μg/g_db_)						
GW1	732.71 ± 0.41	nd	nd	nd	229.21 ± 39.59	244.16 ± 29.77
T10	827.66 ± 8.45	5.28 ± 0.15	9.19 ± 0.15	9.72 ± 0.30	268.69 ± 7.17	230.88 ± 7.47
S10	754.16 ± 4.09	4.93 ± 0.22	9.11 ± 0.90	8.37 ± 0.15	371.44 ± 3.90	216.55 ± 3.00
MD5	959.15 ± 3.04	5.36 ± 0.23	10.41 ± 0.30	11.15 ± 0.15	288.51 ± 1.80	161.04 ± 2.40
X10	854.20 ± 10.44	6.33 ± 0.60	7.60 ± 0.30	7.81 ± 0.00	301.31 ± 8.06	177.58 ± 5.08
3,4-dihidroxybenzoic acid (μg/g_db_)						
GW1	60.22 ± 0.21	nd	nd	nd	136.60 ± 5.66	149.23 ± 9.23
T10	66.10 ± 9.22	nd	nd	nd	106.89 ± 1.79	143.64 ± 2.99
S10	71.63 ± 0.48	nd	nd	nd	65.90 ± 7.49	51.06 ± 0.90
MD5	79.60 ± 0.29	nd	nd	nd	61.82 ± 3.30	33.99 ± 3.00
X10	69.38 ± 2.24	nd	nd	nd	53.21 ± 4.18	52.15 ± 3.28
*p*-hidroxybenzoic acid (μg/g_db_)						
GW1	18.39 ± 1.23	5.00 ± 7.07	6.42 ± 9.08	3.89 ± 5.51	10.73 ± 15.18	8.84 ± 12.50
T10	27.76 ± 2.99	18.75 ± 3.51	25.45 ± 2.54	20.91 ± 2.09	31.47 ± 3.29	27.67 ± 0.30
S10	32.50 ± 0.72	18.86 ± 0.15	24.05 ± 1.35	16.32 ± 0.90	46.40 ± 1.50	37.72 ± 5.99
MD5	28.67 ± 1.25	22.15 ± 2.03	16.68 ± 3.46	15.51 ± 0.60	55.66 ± 3.61	52.05 ± 3.91
X10	14.80 ± 0.32	19.74 ± 2.09	27.13 ± 0.75	22.59 ± 0.30	61.23 ± 4.18	58.49 ± 4.48
Catechin (μg/g_db_)						
GW1	4675.02 ± 99.35	nd	nd	nd	601.56 ± 35.72	701.54 ± 29.47
T10	5928.03 ± 65.47	nd	nd	nd	495.78 ± 1.49	591.47 ± 22.11
S10	4861.45 ± 80.73	nd	nd	nd	593.28 ± 47.35	630.58 ± 47.35
MD5	5325.62 ± 14.85	nd	nd	nd	496.92 ± 12.32	249.84 ± 2.40
X10	4359.75 ± 12.53	nd	nd	nd	459.25 ± 2.09	488.60 ± 37.62
Vanillic acid (μg/g_db_)						
GW1	41.55 ± 0.54	nd	nd	nd	18.94 ± 2.38	22.73 ± 0.00
T10	45.35 ± 0.41	nd	nd	nd	18.80 ± 0.30	18.17 ± 0.60
S10	42.66 ± 0.66	nd	nd	nd	15.26 ± 2.40	16.53 ± 0.00
MD5	47.55 ± 0.24	nd	nd	nd	16.57± 0.00	12.53 ± 0.30
X10	35.28 ± 0.66	nd	nd	nd	17.53 ± 0.30	14.36 ± 0.00
Procyanindin B2 (μg/g_db_)						
GW1	1034.65 ± 105.81	117.08 ± 1.71	nd	24.31 ± 0.74	348.77 ± 2.08	466.01 ± 45.84
T10	1060.26 ± 49.77	110.95 ± 5.60	135.51 ± 2.54	30.84 ± 1.79	57.03 ± 13.74	91.47 ± 8.66
S10	1401.46 ± 31.99	172.48 ± 4.49	130.73 ± 7.19	73.84 ± 12.44	83.91 ± 8.99	85.39 ± 3.90
MD5	1158.63 ± 54.88	131.82 ± 6.16	55.66 ± 12.62	nd	58.85 ± 14.12	37.18 ± 2.70
X10	883.58 ± 41.05	186.97 ± 13.44	119.72 ± 9.26	50.68 ± 3.88	82.98 ± 10.45	61.87 ± 11.05
Epicatechin (μg/g_db_)						
GW1	3284.86 ± 10.78	1228.00 ± 28.58	335.93 ± 5.66	360.35 ± 3.57	152.39 ± 0.60	97.03 ± 4.46
T10	4001.84 ± 38.51	1122.73 ± 107.10	1074.57 ± 44.21	572.88 ± 16.13	336.92 ± 2.09	335.87 ± 27.48
S10	3238.02 ± 50.81	1361.43 ± 23.60	824.03 ± 27.57	570.72 ± 11.84	263.59 ± 10.19	223.96 ± 11.69
MD5	3650.18 ± 5.68	1092.79 ± 74.44	504.14 ± 36.65	334.93 ± 9.16	215.42 ± 14.42	193.97 ± 2.10
X10	2012.11 ± 49.61	1154.77 ± 8.96	657.73 ± 5.37	467.91 ± 0.00	255.70 ± 8.06	266.89 ± 1.79
Galocatechin gallate (μg/g_db_)						
GW1	1229.93 ± 25.65	nd	680.38 ± 16.52	659.44 ± 3.57	2117.66 ± 24.11	2144.18 ± 10.42
T10	1403.32 ± 57.59	nd	812.21 ± 22.11	nd	2050.27 ± 1.79	2188.42 ± 23.90
S10	1475.91 ± 9.08	nd	781.33 ± 10.04	766.82 ± 26.97	2095.56 ± 40.15	2137.09 ± 14.98
MD5	1393.36 ± 7.50	nd	735.29 ± 3.00	779.80 ± 23.28	1951.14 ± 20.43	1840.88 ± 19.53
X10	1259.73 ± 12.22	nd	nd	768.79 ± 24.78	1969.17 ± 53.15	1893.16 ± 38.82
Ferulic acid (μg/g_db_)						
GW1	3.47 ± 0.06	nd	nd	nd	4.21 ± 1.79	11.79 ± 0.00
T10	3.80 ± 0.11	nd	nd	nd	12.04 ± 2.09	16.69 ± 2.09
S10	3.73 ± 0.04	nd	nd	nd	11.23 ± 0.30	14.83 ± 2.40
MD5	4.95 ± 0.15	nd	nd	nd	11.90 ± 0.00	7.65 ± 0.00
X10	4.12 ± 0.58	nd	nd	nd	15.84 ± 0.30	10.77 ± 0.30
Epicatechin gallate (μg/g_db_)						
GW1	168.75 ± 3.24	nd	nd	nd	384.76 ± 4.17	491.69 ± 16.07
T10	221.77 ± 16.62	nd	nd	nd	566.12 ± 2.99	628.64 ± 5.38
S10	184.63 ± 7.28	nd	nd	nd	380.97 ± 28.77	660.24 ± 32.96
MD5	260.80 ± 10.43	nd	nd	nd	217.55 ± 13.82	370.51 ± 12.62
X10	181.83 ± 0.81	nd	nd	nd	553.63 ± 33.44	572.00 ± 19.41
*o*-coumaric acid (μg/g_db_)						
GW1	13.49 ± 0.17	58.62 ± 2.68	15.05 ± 0.74	21.36 ± 0.15	49.67 ± 1.19	98.72 ± 6.85
T10	15.19 ± 0.89	93.42 ± 4.85	161.70 ± 2.54	88.61 ± 1.05	48.16 ± 4.78	71.61 ± 2.69
S10	12.78 ± 0.58	116.91 ± 0.22	102.77 ± 0.30	34.54 ± 2.70	63.14 ± 0.60	50.01 ± 4.79
MD5	14.87 ± 0.39	75.69 ± 4.73	18.91 ± 3.00	7.75 ± 1.95	42.28 ± 0.30	38.03 ± 0.30
X10	12.64 ± 1.57	80.82 ± 3.06	44.76 ± 3.88	36.95 ± 0.00	59.12 ± 2.99	50.46 ± 2.09

OP—oral phase, GP—gastric phase, IP—intestinal phase, nd—not detected. Index numbers associated with abbreviations indicate the time interval when a certain sample was taken (i.e., OP_3_—3rd minute of the oral phase). Phenolic contents are expressed as mean values (μg/g_db_) ± SDs.

## Data Availability

The original contributions presented in the study are included in the article, further inquiries can be directed to the corresponding authors.
